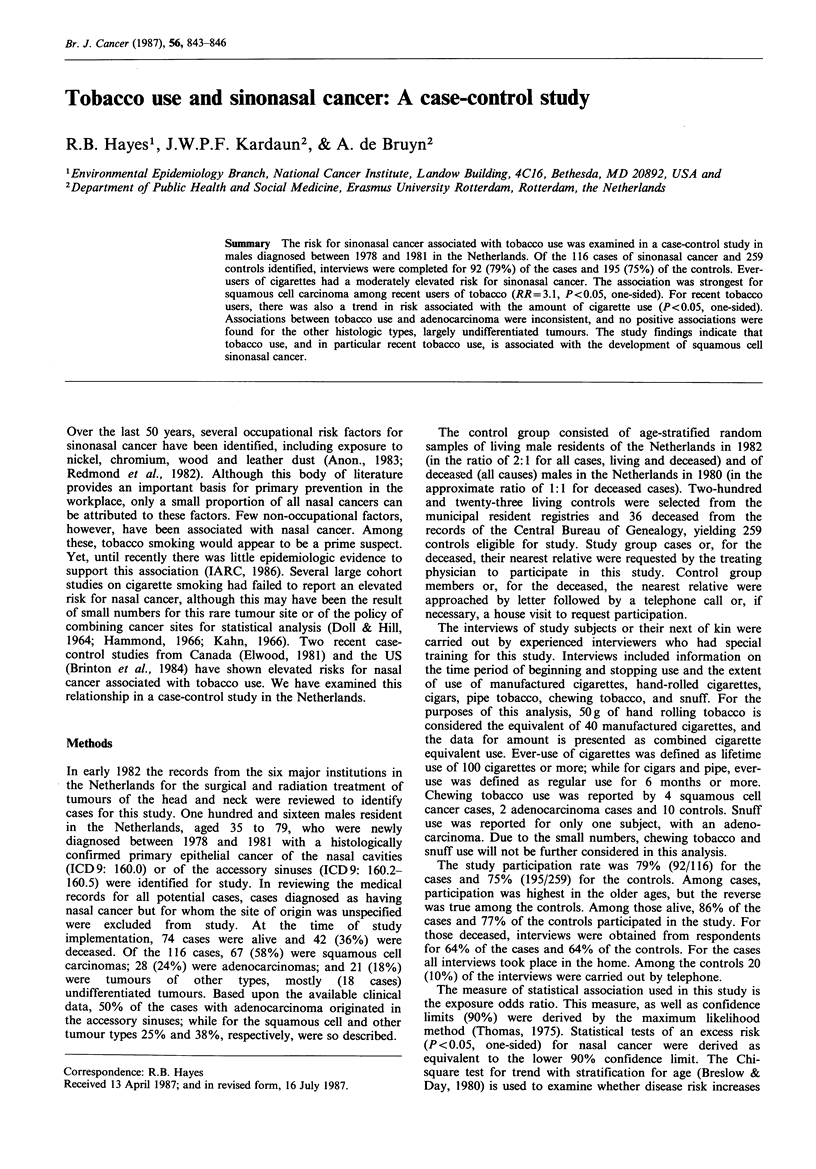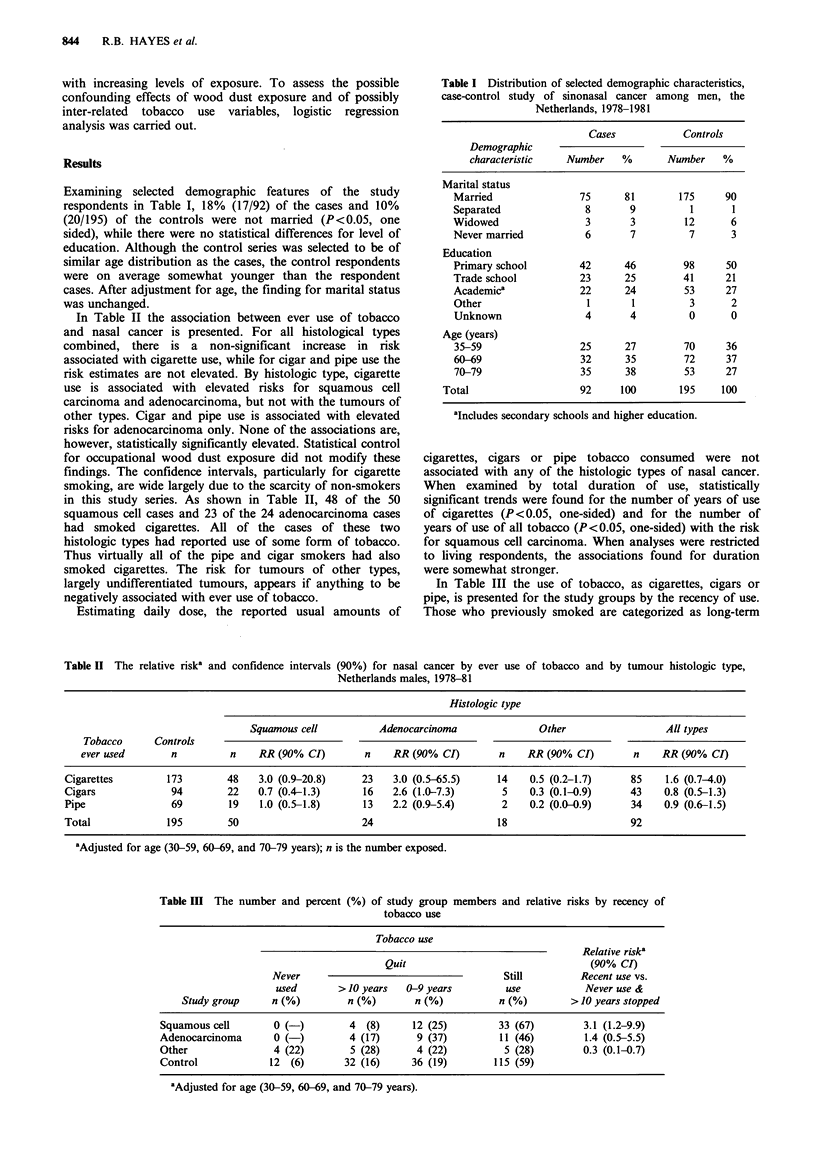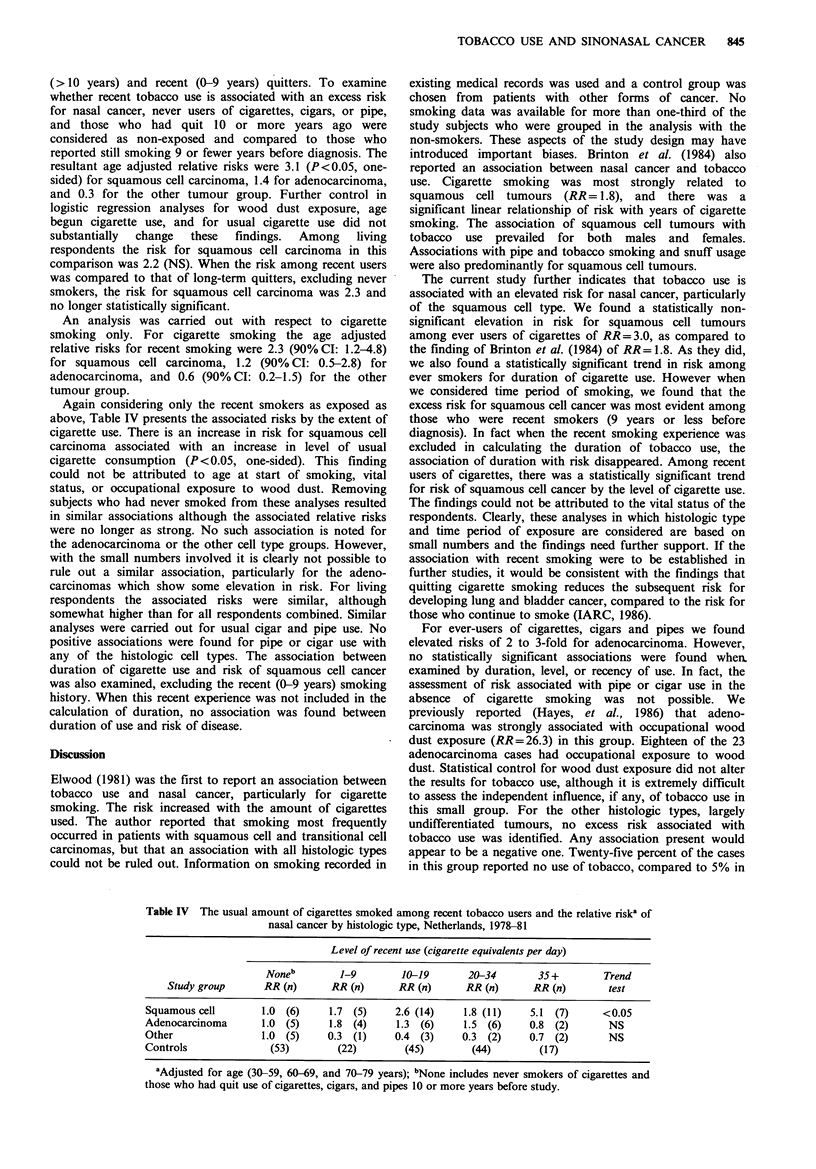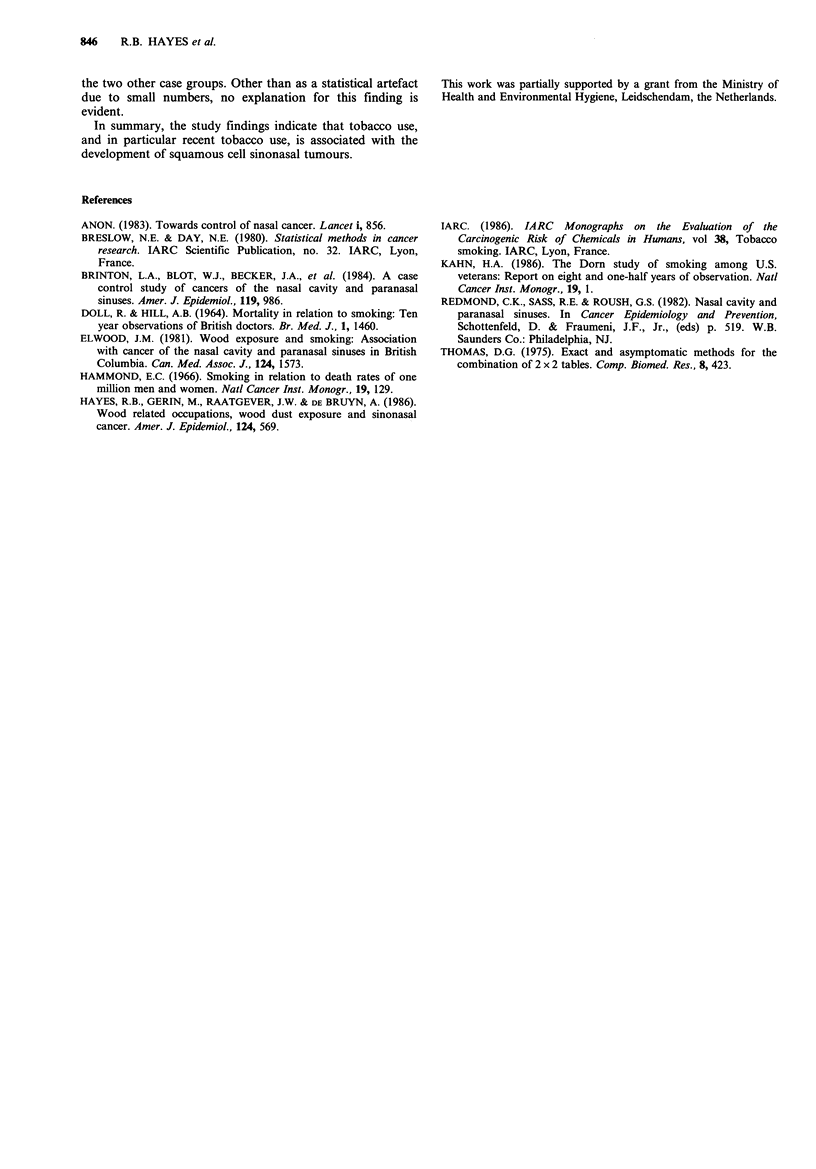# Tobacco use and sinonasal cancer: a case-control study.

**DOI:** 10.1038/bjc.1987.303

**Published:** 1987-12

**Authors:** R. B. Hayes, J. W. Kardaun, A. de Bruyn

**Affiliations:** Environmental Epidemiology Branch, National Cancer Institute, Bethesda, MD 20892.

## Abstract

The risk for sinonasal cancer associated with tobacco use was examined in a case-control study in males diagnosed between 1978 and 1981 in the Netherlands. Of the 116 cases of sinonasal cancer and 259 controls identified, interviews were completed for 92 (79%) of the cases and 195 (75%) of the controls. Ever-users of cigarettes had a moderately elevated risk for sinonasal cancer. The association was strongest for squamous cell carcinoma among recent users of tobacco (RR = 3.1, P less than 0.05, one-sided). For recent tobacco users, there was also a trend in risk associated with the amount of cigarette use (P less than 0.05, one-sided). Associations between tobacco use and adenocarcinoma were inconsistent, and no positive associations were found for the other histologic types, largely undifferentiated tumours. The study findings indicate that tobacco use, and in particular recent tobacco use, is associated with the development of squamous cell sinonasal cancer.


Br. J. Cancer (1987), 56, 843–846

### Tobacco use and sinonasal cancer: A case-control study

R.B. Hayes[1], J.W.P.F. Kardaun[2], & A. de Bruyn[2]

[1]*Environmental Epidemiology Branch, National Cancer Institute, Landow Building, 4C16, Bethesda, MD 20892, USA and* [2]*Department of Public Health and Social Medicine, Erasmus University Rotterdam, Rotterdam, the Netherlands*

**Summary** The risk for sinonasal cancer associated with tobacco use was examined in a case-control study in males diagnosed between 1978 and 1981 in the Netherlands. Of the 116 cases of sinonasal cancer and 259 controls identified, interviews were completed for 92 (79%) of the cases and 195 (75%) of the controls. Ever-users of cigarettes had a moderately elevated risk for sinonasal cancer. The association was strongest for squamous cell carcinoma among recent users of tobacco ($RR=3.1$, $P<0.05$, one-sided). For recent tobacco users, there was also a trend in risk associated with the amount of cigarette use ($P<0.05$, one-sided). Associations between tobacco use and adenocarcinoma were inconsistent, and no positive associations were found for the other histologic types, largely undifferentiated tumours. The study findings indicate that tobacco use, and in particular recent tobacco use, is associated with the development of squamous cell sinonasal cancer.

Over the last 50 years, several occupational risk factors for sinonasal cancer have been identified, including exposure to nickel, chromium, wood and leather dust (Anon., 1983; Redmond *et al.*, 1982). Although this body of literature provides an important basis for primary prevention in the workplace, only a small proportion of all nasal cancers can be attributed to these factors. Few non-occupational factors, however, have been associated with nasal cancer. Among these, tobacco smoking would appear to be a prime suspect. Yet, until recently there was little epidemiologic evidence to support this association (IARC, 1986). Several large cohort studies on cigarette smoking had failed to report an elevated risk for nasal cancer, although this may have been the result of small numbers for this rare tumour site or of the policy of combining cancer sites for statistical analysis (Doll & Hill, 1964; Hammond, 1966; Kahn, 1966). Two recent case-control studies from Canada (Elwood, 1981) and the US (Brinton *et al.*, 1984) have shown elevated risks for nasal cancer associated with tobacco use. We have examined this relationship in a case-control study in the Netherlands.

### Methods

In early 1982 the records from the six major institutions in the Netherlands for the surgical and radiation treatment of tumours of the head and neck were reviewed to identify cases for this study. One hundred and sixteen males resident in the Netherlands, aged 35 to 79, who were newly diagnosed between 1978 and 1981 with a histologically confirmed primary epithelial cancer of the nasal cavities (ICD9: 160.0) or of the accessory sinuses (ICD9: 160.2–160.5) were identified for study. In reviewing the medical records for all potential cases, cases diagnosed as having nasal cancer but for whom the site of origin was unspecified were excluded from study. At the time of study implementation, 74 cases were alive and 42 (36%) were deceased. Of the 116 cases, 67 (58%) were squamous cell carcinomas; 28 (24%) were adenocarcinomas; and 21 (18%) were tumours of other types, mostly (18 cases) undifferentiated tumours. Based upon the available clinical data, 50% of the cases with adenocarcinoma originated in the accessory sinuses; while for the squamous cell and other tumour types 25% and 38%, respectively, were so described.

Correspondence: R.B. Hayes
Received 13 April 1987; and in revised form, 16 July 1987.

The control group consisted of age-stratified random samples of living male residents of the Netherlands in 1982 (in the ratio of 2:1 for all cases, living and deceased) and of deceased (all causes) males in the Netherlands in 1980 (in the approximate ratio of 1:1 for deceased cases). Two-hundred and twenty-three living controls were selected from the municipal resident registries and 36 deceased from the records of the Central Bureau of Genealogy, yielding 259 controls eligible for study. Study group cases or, for the deceased, their nearest relative were requested by the treating physician to participate in this study. Control group members or, for the deceased, the nearest relative were approached by letter followed by a telephone call or, if necessary, a house visit to request participation.

The interviews of study subjects or their next of kin were carried out by experienced interviewers who had special training for this study. Interviews included information on the time period of beginning and stopping use and the extent of use of manufactured cigarettes, hand-rolled cigarettes, cigars, pipe tobacco, chewing tobacco, and snuff. For the purposes of this analysis, 50 g of hand rolling tobacco is considered the equivalent of 40 manufactured cigarettes, and the data for amount is presented as combined cigarette equivalent use. Ever-use of cigarettes was defined as lifetime use of 100 cigarettes or more; while for cigars and pipe, ever-use was defined as regular use for 6 months or more. Chewing tobacco use was reported by 4 squamous cell cancer cases, 2 adenocarcinoma cases and 10 controls. Snuff use was reported for only one subject, with an adenocarcinoma. Due to the small numbers, chewing tobacco and snuff use will not be further considered in this analysis.

The study participation rate was 79% (92/116) for the cases and 75% (195/259) for the controls. Among cases, participation was highest in the older ages, but the reverse was true among the controls. Among those alive, 86% of the cases and 77% of the controls participated in the study. For those deceased, interviews were obtained from respondents for 64% of the cases and 64% of the controls. For the cases all interviews took place in the home. Among the controls 20 (10%) of the interviews were carried out by telephone.

The measure of statistical association used in this study is the exposure odds ratio. This measure, as well as confidence limits (90%) were derived by the maximum likelihood method (Thomas, 1975). Statistical tests of an excess risk ($P<0.05$, one-sided) for nasal cancer were derived as equivalent to the lower 90% confidence limit. The Chi-square test for trend with stratification for age (Breslow & Day, 1980) is used to examine whether disease risk increases with increasing levels of exposure. To assess the possible confounding effects of wood dust exposure and of possibly inter-related tobacco use variables, logistic regression analysis was carried out.

### Results

Examining selected demographic features of the study respondents in Table I, 18% (17/92) of the cases and 10% (20/195) of the controls were not married ($P<0.05$, one sided), while there were no statistical differences for level of education. Although the control series was selected to be of similar age distribution as the cases, the control respondents were on average somewhat younger than the respondent cases. After adjustment for age, the finding for marital status was unchanged.

In Table II the association between ever use of tobacco and nasal cancer is presented. For all histological types combined, there is a non-significant increase in risk associated with cigarette use, while for cigar and pipe use the risk estimates are not elevated. By histologic type, cigarette use is associated with elevated risks for squamous cell carcinoma and adenocarcinoma, but not with the tumours of other types. Cigar and pipe use is associated with elevated risks for adenocarcinoma only. None of the associations are, however, statistically significantly elevated. Statistical control for occupational wood dust exposure did not modify these findings. The confidence intervals, particularly for cigarette smoking, are wide largely due to the scarcity of non-smokers in this study series. As shown in Table II, 48 of the 50 squamous cell cases and 23 of the 24 adenocarcinoma cases had smoked cigarettes. All of the cases of these two histologic types had reported use of some form of tobacco. Thus virtually all of the pipe and cigar smokers had also smoked cigarettes. The risk for tumours of other types, largely undifferentiated tumours, appears if anything to be negatively associated with ever use of tobacco.

Estimating daily dose, the reported usual amounts of cigarettes, cigars or pipe tobacco consumed were not associated with any of the histologic types of nasal cancer. When examined by total duration of use, statistically significant trends were found for the number of years of use of cigarettes ($P<0.05$, one-sided) and for the number of years of use of all tobacco ($P<0.05$, one-sided) with the risk for squamous cell carcinoma. When analyses were restricted to living respondents, the associations found for duration were somewhat stronger.

In Table III the use of tobacco, as cigarettes, cigars or pipe, is presented for the study groups by the recency of use. Those who previously smoked are categorized as long-term

**Table I** Distribution of selected demographic characteristics, case-control study of sinonasal cancer among men, the Netherlands, 1978–1981

| *Demographic characteristic* | *Cases* Number | % | *Controls* Number | % |
|---|---|---|---|---|
| Marital status | | | | |
| Married | 75 | 81 | 175 | 90 |
| Separated | 8 | 9 | 1 | 1 |
| Widowed | 3 | 3 | 12 | 6 |
| Never married | 6 | 7 | 7 | 3 |
| Education | | | | |
| Primary school | 42 | 46 | 98 | 50 |
| Trade school | 23 | 25 | 41 | 21 |
| Academic[a] | 22 | 24 | 53 | 27 |
| Other | 1 | 1 | 3 | 2 |
| Unknown | 4 | 4 | 0 | 0 |
| Age (years) | | | | |
| 35–59 | 25 | 27 | 70 | 36 |
| 60–69 | 32 | 35 | 72 | 37 |
| 70–79 | 35 | 38 | 53 | 27 |
| Total | 92 | 100 | 195 | 100 |

[a]Includes secondary schools and higher education.

**Table II** The relative risk[a] and confidence intervals (90%) for nasal cancer by ever use of tobacco and by tumour histologic type, Netherlands males, 1978–81

| *Tobacco ever used* | *Controls n* | *Squamous cell* *n* | *RR (90% CI)* | *Adenocarcinoma* *n* | *RR (90% CI)* | *Other* *n* | *RR (90% CI)* | *All types* *n* | *RR (90% CI)* |
|---|---|---|---|---|---|---|---|---|---|
| Cigarettes | 173 | 48 | 3.0 (0.9–20.8) | 23 | 3.0 (0.5–65.5) | 14 | 0.5 (0.2–1.7) | 85 | 1.6 (0.7–4.0) |
| Cigars | 94 | 22 | 0.7 (0.4–1.3) | 16 | 2.6 (1.0–7.3) | 5 | 0.3 (0.1–0.9) | 43 | 0.8 (0.5–1.3) |
| Pipe | 69 | 19 | 1.0 (0.5–1.8) | 13 | 2.2 (0.9–5.4) | 2 | 0.2 (0.0–0.9) | 34 | 0.9 (0.6–1.5) |
| Total | 195 | 50 | | 24 | | 18 | | 92 | |

[a]Adjusted for age (30–59, 60–69, and 70–79 years); *n* is the number exposed.

**Table III** The number and percent (%) of study group members and relative risks by recency of tobacco use

| *Study group* | *Never used n (%)* | *Quit >10 years n (%)* | *Quit 0–9 years n (%)* | Still use *n (%)* | *Relative risk[a] (90% CI) Recent use* vs. *Never use & >10 years stopped* |
|---|---|---|---|---|---|
| Squamous cell | 0 (—) | 4 (8) | 12 (25) | 33 (67) | 3.1 (1.2–9.9) |
| Adenocarcinoma | 0 (—) | 4 (17) | 9 (37) | 11 (46) | 1.4 (0.5–5.5) |
| Other | 4 (22) | 5 (28) | 4 (22) | 5 (28) | 0.3 (0.1–0.7) |
| Control | 12 (6) | 32 (16) | 36 (19) | 115 (59) | |

[a]Adjusted for age (30–59, 60–69, and 70–79 years).

(>10 years) and recent (0–9 years) quitters. To examine whether recent tobacco use is associated with an excess risk for nasal cancer, never users of cigarettes, cigars, or pipe, and those who had quit 10 or more years ago were considered as non-exposed and compared to those who reported still smoking 9 or fewer years before diagnosis. The resultant age adjusted relative risks were 3.1 ($P<0.05$, one-sided) for squamous cell carcinoma, 1.4 for adenocarcinoma, and 0.3 for the other tumour group. Further control in logistic regression analyses for wood dust exposure, age begun cigarette use, and for usual cigarette use did not substantially change these findings. Among living respondents the risk for squamous cell carcinoma in this comparison was 2.2 (NS). When the risk among recent users was compared to that of long-term quitters, excluding never smokers, the risk for squamous cell carcinoma was 2.3 and no longer statistically significant.

An analysis was carried out with respect to cigarette smoking only. For cigarette smoking the age adjusted relative risks for recent smoking were 2.3 (90% CI: 1.2–4.8) for squamous cell carcinoma, 1.2 (90% CI: 0.5–2.8) for adenocarcinoma, and 0.6 (90% CI: 0.2–1.5) for the other tumour group.

Again considering only the recent smokers as exposed as above, Table IV presents the associated risks by the extent of cigarette use. There is an increase in risk for squamous cell carcinoma associated with an increase in level of usual cigarette consumption ($P<0.05$, one-sided). This finding could not be attributed to age at start of smoking, vital status, or occupational exposure to wood dust. Removing subjects who had never smoked from these analyses resulted in similar associations although the associated relative risks were no longer as strong. No such association is noted for the adenocarcinoma or the other cell type groups. However, with the small numbers involved it is clearly not possible to rule out a similar association, particularly for the adenocarcinomas which show some elevation in risk. For living respondents the associated risks were similar, although somewhat higher than for all respondents combined. Similar analyses were carried out for usual cigar and pipe use. No positive associations were found for pipe or cigar use with any of the histologic cell types. The association between duration of cigarette use and risk of squamous cell cancer was also examined, excluding the recent (0–9 years) smoking history. When this recent experience was not included in the calculation of duration, no association was found between duration of use and risk of disease.

### Discussion

Elwood (1981) was the first to report an association between tobacco use and nasal cancer, particularly for cigarette smoking. The risk increased with the amount of cigarettes used. The author reported that smoking most frequently occurred in patients with squamous cell and transitional cell carcinomas, but that an association with all histologic types could not be ruled out. Information on smoking recorded in existing medical records was used and a control group was chosen from patients with other forms of cancer. No smoking data was available for more than one-third of the study subjects who were grouped in the analysis with the non-smokers. These aspects of the study design may have introduced important biases. Brinton *et al.* (1984) also reported an association between nasal cancer and tobacco use. Cigarette smoking was most strongly related to squamous cell tumours ($RR=1.8$), and there was a significant linear relationship of risk with years of cigarette smoking. The association of squamous cell tumours with tobacco use prevailed for both males and females. Associations with pipe and tobacco smoking and snuff usage were also predominantly for squamous cell tumours.

The current study further indicates that tobacco use is associated with an elevated risk for nasal cancer, particularly of the squamous cell type. We found a statistically non-significant elevation in risk for squamous cell tumours among ever users of cigarettes of $RR=3.0$, as compared to the finding of Brinton *et al.* (1984) of $RR=1.8$. As they did, we also found a statistically significant trend in risk among ever smokers for duration of cigarette use. However when we considered time period of smoking, we found that the excess risk for squamous cell cancer was most evident among those who were recent smokers (9 years or less before diagnosis). In fact when the recent smoking experience was excluded in calculating the duration of tobacco use, the association of duration with risk disappeared. Among recent users of cigarettes, there was a statistically significant trend for risk of squamous cell cancer by the level of cigarette use. The findings could not be attributed to the vital status of the respondents. Clearly, these analyses in which histologic type and time period of exposure are considered are based on small numbers and the findings need further support. If the association with recent smoking were to be established in further studies, it would be consistent with the findings that quitting cigarette smoking reduces the subsequent risk for developing lung and bladder cancer, compared to the risk for those who continue to smoke (IARC, 1986).

For ever-users of cigarettes, cigars and pipes we found elevated risks of 2 to 3-fold for adenocarcinoma. However, no statistically significant associations were found when examined by duration, level, or recency of use. In fact, the assessment of risk associated with pipe or cigar use in the absence of cigarette smoking was not possible. We previously reported (Hayes, *et al.*, 1986) that adenocarcinoma was strongly associated with occupational wood dust exposure ($RR=26.3$) in this group. Eighteen of the 23 adenocarcinoma cases had occupational exposure to wood dust. Statistical control for wood dust exposure did not alter the results for tobacco use, although it is extremely difficult to assess the independent influence, if any, of tobacco use in this small group. For the other histologic types, largely undifferentiated tumours, no excess risk associated with tobacco use was identified. Any association present would appear to be a negative one. Twenty-five percent of the cases in this group reported no use of tobacco, compared to 5% in

**Table IV** The usual amount of cigarettes smoked among recent tobacco users and the relative risk[a] of nasal cancer by histologic type, Netherlands, 1978–81

| | *Level of recent use (cigarette equivalents per day)* | | | | | |
|---|---|---|---|---|---|---|
| *Study group* | *None*[b] *RR (n)* | *1–9 RR (n)* | *10–19 RR (n)* | *20–34 RR (n)* | *35+ RR (n)* | *Trend test* |
| Squamous cell | 1.0 (6) | 1.7 (5) | 2.6 (14) | 1.8 (11) | 5.1 (7) | <0.05 |
| Adenocarcinoma | 1.0 (5) | 1.8 (4) | 1.3 (6) | 1.5 (6) | 0.8 (2) | NS |
| Other | 1.0 (5) | 0.3 (1) | 0.4 (3) | 0.3 (2) | 0.7 (2) | NS |
| Controls | (53) | (22) | (45) | (44) | (17) | |

[a]Adjusted for age (30–59, 60–69, and 70–79 years); [b]None includes never smokers of cigarettes and those who had quit use of cigarettes, cigars, and pipes 10 or more years before study.

the two other case groups. Other than as a statistical artefact due to small numbers, no explanation for this finding is evident.

In summary, the study findings indicate that tobacco use, and in particular recent tobacco use, is associated with the development of squamous cell sinonasal tumours.

This work was partially supported by a grant from the Ministry of Health and Environmental Hygiene, Leidschendam, the Netherlands.

### References

ANON. (1983). Towards control of nasal cancer. *Lancet* **i**, 856.

BRESLOW, N.E. & DAY, N.E. (1980). *Statistical methods in cancer research*. IARC Scientific Publication, no. 32. IARC, Lyon, France.

BRINTON, L.A., BLOT, W.J., BECKER, J.A., *et al.* (1984). A case control study of cancers of the nasal cavity and paranasal sinuses. *Amer. J. Epidemiol.*, **119**, 986.

DOLL, R. & HILL, A.B. (1964). Mortality in relation to smoking: Ten year observations of British doctors. *Br. Med. J.*, **1**, 1460.

ELWOOD, J.M. (1981). Wood exposure and smoking: Association with cancer of the nasal cavity and paranasal sinuses in British Columbia. *Can. Med. Assoc. J.*, **124**, 1573.

HAMMOND, E.C. (1966). Smoking in relation to death rates of one million men and women. *Natl Cancer Inst. Monogr.*, **19**, 129.

HAYES, R.B., GERIN, M., RAATGEVER, J.W. & DE BRUYN, A. (1986). Wood related occupations, wood dust exposure and sinonasal cancer. *Amer. J. Epidemiol.*, **124**, 569.

IARC. (1986). *IARC Monographs on the Evaluation of the Carcinogenic Risk of Chemicals in Humans*, vol **38**, Tobacco smoking. IARC, Lyon, France.

KAHN, H.A. (1986). The Dorn study of smoking among U.S. veterans: Report on eight and one-half years of observation. *Natl Cancer Inst. Monogr.*, **19**, 1.

REDMOND, C.K., SASS, R.E. & ROUSH, G.S. (1982). Nasal cavity and paranasal sinuses. In *Cancer Epidemiology and Prevention*, Schottenfeld, D. & Fraumeni, J.F., Jr., (eds) p. 519. W.B. Saunders Co.: Philadelphia, NJ.

THOMAS, D.G. (1975). Exact and asymptomatic methods for the combination of 2 × 2 tables. *Comp. Biomed. Res.*, **8**, 423.